# Exercises for Weak Feet

**Published:** 1901-11-23

**Authors:** 


### EXERCISES FOR WEAK FEET.

Dr. Henry Ling Taylor,[1] of New York, says that putting on one side all cases of deformities due to paralysis, rickets, and other gross organic affections, and also cases of fully-developed flat foot, cases are frequently met with which we may properly group together as "weak feet." Children so affected seldom have pain, but their gait is awkward and inelastic. In treating this condition, which from his description we should certainly class as an early stage of flat foot, he says attention should be paid to the shoes, which should be heel-less, narrow at the back, broad in front, and straight on the inner side. In addition, if the inner ankle projects abnormally, the shoes should be raised the whole length of the inner side by a wedgeshaped piece of sole leather a quarter of an inch thick at the inner edge, as recommended by the late Mr. Thomas, and the child should be trained to walk with the inner edges of the feet parallel or slightly turned in. Thus the weight is thrown on the outer edge where it ought to fall. "Children should be trained in schools and gymnasiums to walk and stand with nearly parallel feet—the whole race of dancing masters, physical trainers, and military experts to the contrary notwithstanding." Having provided proper shoes and trained the child to toe straight, or slightly inwards, the next step is to strengthen and train the muscles, especially the tibials, which invert the foot. This is done by special exercises with the shoes off night and morning. Some of these consist of movements of adduction and inversion of the feet while seated, others of 'heel raising,' or rising on the balls of the feet, while the patient stands, toeing in, with the hands on the table to balance the body. If the patient is old enough, this is best done in four counts, starting with the feet parallel. (1) Raise heels as far as possible; (2) turn heels outwards; (3) turn heels inwards; (4) lower heels. All the movements should be done very slowly with straight knees, and repeated 10 or 20 times. It is much better to do them a few times correctly than many times carelessly. The patient should also practise walking and trotting, with heels raised and toes slightly turned in, and also walking on the outer borders of the feet.

[1] Journal of Physical Therapeutics, Oct.

